# A role for BMP-induced homeobox gene *MIXL1* in acute myelogenous leukemia and identification of type I BMP receptor as a potential target for therapy

**DOI:** 10.18632/oncotarget.2564

**Published:** 2014-12-17

**Authors:** Aaron Raymond, Bin Liu, Hong Liang, Ciamaio Wei, Michele Guindani, Yue Lu, Shoudan Liang, Lisa S. St. John, Jeff Molldrem, Lalitha Nagarajan

**Affiliations:** ^1^ Department of Genetics, the University of Texas MD Anderson Cancer Center, Houston, TX 77030, USA; ^2^ Graduate Program in Genes and Development, the University of Texas MD Anderson Cancer Center, Houston, TX 77030, USA; ^3^ Center for Cancer Genetics and Genomics, the University of Texas MD Anderson Cancer Center, Houston, TX 77030, USA; ^4^ Department of Biostatistics, the University of Texas MD Anderson Cancer Center, Houston, TX 77030, USA; ^5^ Dept. of Leukemia, the University of Texas MD Anderson Cancer Center, Houston, TX 77030, USA; ^6^ Dept. of Molecular Carcinogenesis, the University of Texas MD Anderson Cancer Center, Houston, TX 77030, USA; ^7^ Dept. of Bioinformatics and Computational Biology, the University of Texas MD Anderson Cancer Center, Houston, TX 77030, USA; ^8^ Section of Transplantation Immunology, Department of Stem Cell Transplantation and Cellular Therapy, the University of Texas MD Anderson Cancer Center, Houston, TX 77030, USA; ^9^ Graduate Program in Human Molecular Genetics, the University of Texas MD Anderson Cancer Center, Houston, TX 77030, USA; ^10^ Center for Stem cell and Developmental biology, the University of Texas MD Anderson Cancer Center, Houston, TX 77030, USA

## Abstract

*Mesoderm Inducer in Xenopus Like1 (MIXL1)*, a paired-type homeobox transcription factor induced by TGF-β family of ligands is required for early embryonic specification of mesoderm and endoderm. Retrovirally transduced *Mixl1* is reported to induce acute myelogenous leukemia (AML) with a high penetrance. But the mechanistic underpinnings of *MIXL1* mediated leukemogenesis are unknown. Here, we establish the protooncogene *c-REL* to be a transcriptional target of MIXL1 by genome wide chromatin immune precipitation. Accordingly, expression of *c-REL* and its downstream targets *BCL2L1* and *BCL2A2* are elevated in MIXL1 expressing cells. Notably, MIXL1 regulates *c-REL* through a zinc finger binding motif, potentially by a MIXL1–Zinc finger protein transcriptional complex. Furthermore, MIXL1 expression is detected in the cancer genome atlas (TCGA) AML samples in a pattern mutually exclusive from that of *HOXA9*, *CDX2* and *HLX* suggesting the existence of a core, yet distinct HOX transcriptional program. Finally, we demonstrate *MIXL1* to be induced by BMP4 and not TGF-β in primary human hematopoietic stem and progenitor cells. Consequently, MIXL1 expressing AML cells are preferentially sensitive to the BMPR1 kinase inhibitor LDN-193189. These findings support the existence of a novel *MIXL1-c REL* mediated survival axis in AML that can be targeted by BMPR1 inhibitors. (*MIXL1*- human gene, *Mixl1*- mouse ortholog, MIXL1- protein)

## INTRODUCTION

Acute myelogenous leukemia (AML), the most common leukemia in adults, is clinically and genetically diverse [1]. Overall prognosis of AML remains dismal despite the incremental progress in defining subsets responsive to aggressive chemotherapy [2]. Recent high-throughput genome sequencing efforts have identified several somatic mutations some recurrent and some unique to individual leukemia, uncovering the vast genetic heterogeneity in AML [3–9]. Even as valuable clues emerge from the mutational landscape, challenges remain in discerning therapeutic vulnerabilities. Better understanding of differentially expressed regulatory genes may yield clues on novel target identification. In this regard, homeobox genes (HOX), are constitutively expressed in AML in contrast to the temporal regulation in normal hematopoiesis [10–16]. But HOX proteins remain unexplored as therapeutic targets due to the technical limitations in inhibiting transcription factors.

*MIXL1* the human ortholog of *Mix.1*, a paired-type, non-clustered HOX transcription factor originally isolated in *Xenopus* laevis is aberrantly expressed in AML and lymphomas [17, 18]. In normal homeostasis, *MIXL1* expression is restricted to hematopoietic stem and progenitor cells (HSPCs) [17]. Retroviral transduction of *Mixl1* the mouse ortholog, results in transplantable AML in 100% of mice, suggesting a leukemogenic potential for *Mixl1 [19]*. Additionally, forced expression of *Mixl1* in hematopoietic stem cells confers abnormal, growth factor–dependent self-renewal potential to granulocytic precursors [20]. Over expression of *Mixl1* in mouse embryonic stem cells promotes mesodermal, hemangioblastic, and hematopoietic progenitors consistent with a role for mesoderm induction [21].

*Mix.1* and orthologs of *Mix.1* are induced by TGF-β/BMP family of structurally related secreted molecules [22–24]. In mammals, the TGF-β/BMP family comprises of 24 ligands. The transmemebrane receptor complex consists of two molecules each of type II and type I receptor. Upon ligand binding, type II receptor phosphorylates type I receptor which in turn phosphorylates SMAD transcription factors. Phosphorylated SMADs regulate target gene expression in the nucleus to elicit a growth or differentiation response. Given the functional overlap and redundancy between the ligands [25], it is likely that *Mixl1* may be induced by different ligands in a cell type specific manner. Thus in mouse ES cells TGF-β stimulation results in SMAD 2 and 3 binding to Mixl1 promoter [26]. In hematopoiesis, TGF-β confers quiescence to hematopoietic stem cells (HSCs) raising the possibility that *MIXL1* may not be TGF-β inducible in HSCs [27].

The present study was aimed at determining factors upstream and downstream of *MIXL1* in hematopoiesis and the potential role of *MIXL1* in AML pathogenesis. We identified several transcriptional targets of MIXL1 in myeloid leukemic lines using genome wide chromatin immunoprecipitation. We establish the proto-oncogene *c-REL* to be an important MIXL1 transcriptional target that confers an anti apoptotic advantage to *MIXL1* expressing cells. Upstream of *MIXL1*, BMP4 induces *MIXL1* in HSPCs. Consistent with the BMP mediated induction, AML cells that express *MIXL1* are preferentially sensitive to type 1 BMP/activin receptor kinase inhibition. Together, these results indicate for the first time a novel survival mechanism conferred by *BMP-MIXL1- c-REL* axis in AML which can be targeted by type I BMP receptor kinase inhibitors.

## RESULTS

### Generation of *MIXL1*-expressing AML cell lines

**Figure 1:**
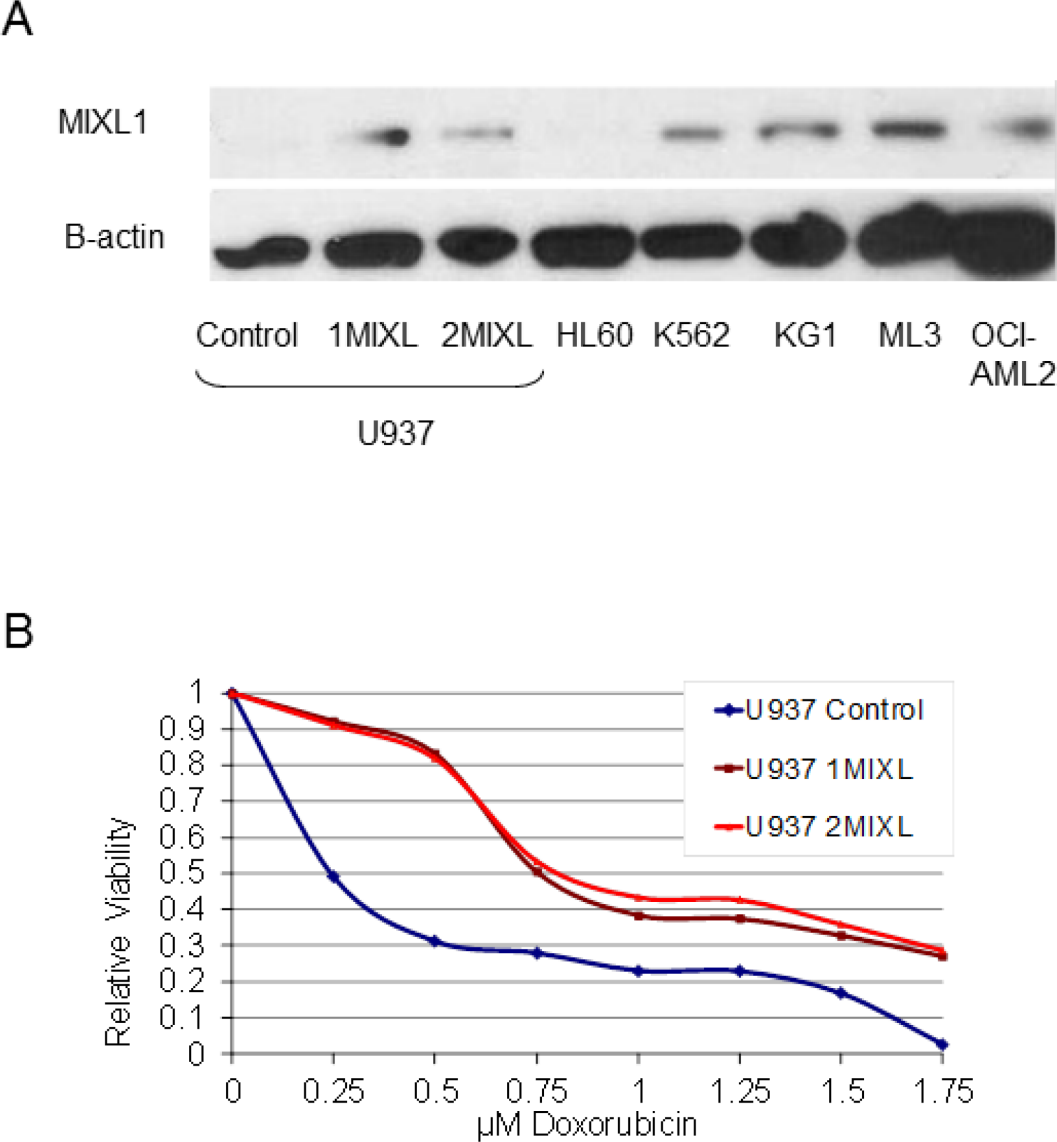
*MIXL1* expression confers decreased sensitivity to doxorubicin in AML cells **(A)** Stable transfectants of U937 cells express MIXL1 at levels similar to those of endogenous MIXL1 in AML cell lines. MIXL1 was detected by probing 30 μg of whole cell lysates resolved on SDS-PAGE and transferred to PVDF membrane, with rabbit antibodies against N-terminal epitope of MIXL1 and with β-actin for a loading control [17]. **(B)** MIXL1 expression reduces sensitivity of U937 cells to doxorubicin. The cell lines were treated with 0–1.75 μM doxorubicin on day 0. Cell survival was measured at 24 hours by MTS assay as detailed in Materials and Methods. Absorbance of untreated cells was normalized to 1. Relative viability at varying concentrations of doxorubicin is denoted.

*MIXL1* expression is varied in AML cell lines. KG1, ML3, and K562 express abundant *MIXL1*, whereas HL60 and U937 do not [17]. The lack of *MIXL1* expression in U937 cells allowed us to generate isogenic cell lines with MIXL1 expression as ectopic expression of transcription factors in these cells has been valuable in elucidation of target genes and pathways for *SET-CAN*, *MLL, MN1* [28–30] Therefore, we established two clonal lines (1MIXL1 and 2MIXL1) expressing HA- FLAG epitope tagged MIXL1 and a control vector–transduced clone in U937 cells. As shown in Figure 1A, *MIXL1* expression levels in the clonal lines were similar to endogenous *MIXL1* levels in K562, KG1, ML3, and OCI-AML2 cells. There were no significant differences in doubling time as measured by conventional MTS assay or clonogenicity in methyl cellulose between the control cells and the 1MIXL1 and 2MIXL1 cells However, response to the alkylating agent doxorubicin differed significantly between the control and MIXL1 expressing cells (Fig. 1B). After 24 hours of treatment, doxorubicin had an LD_50_ of 0.25 μM for the control line and 0.75 μM for 1MIXL and 2MIXL. At 1.75 μM, doxorubicin was cytotoxic to 100% of control cells, whereas 30% of the *MIXL1*-expressing clones appear to survive. These results suggested that *MIXL1* expression confers a survival advantage, potentially through an anti apototic pathway. Importantly, such a subtle yet functional response supported the use of these clonal lines for further characterization of downstream transcriptional targets.

### *c-REL*, a direct transcriptional target of MIXL1

**Figure 2:**
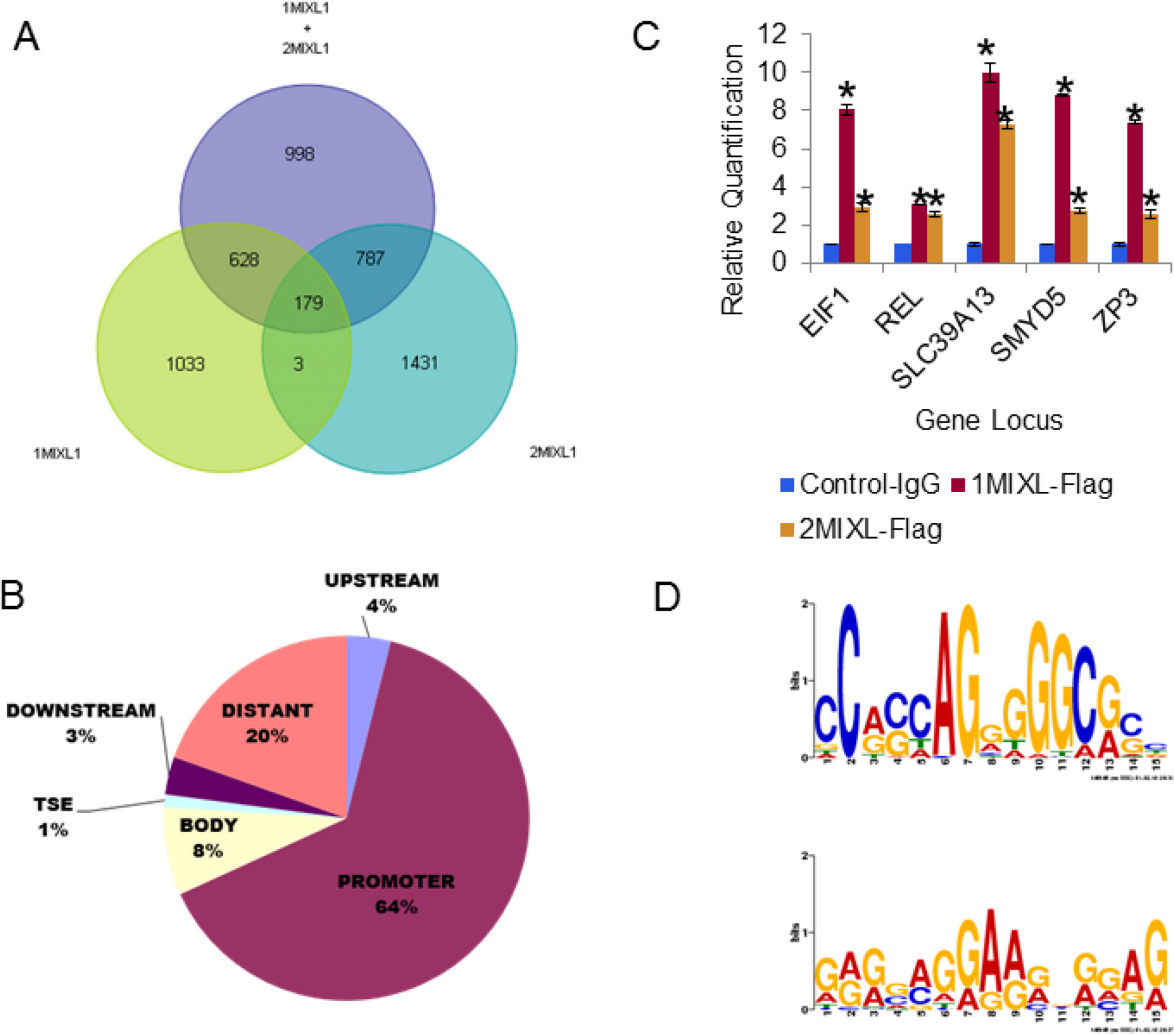
Identification of direct MIXL1 transcriptional targets by ChIP-Sequencing **(A)** Venn diagram 1MIXL-Flag normalized to control-Flag and control-IgG (Set 1), 2MIXL-Flag normalized to control-Flag and control-IgG (Set 2), and 1MIXL-Flag and 2MIXL-Flag combined and normalized to control-Flag and control-IgG (Set 3). A total of 179 peaks shared by the three groups is denoted. **(B)** Pie chart depicting localization of MIXL1 in the human genome. Peaks were classified according to distance from the nearest transcribed gene using the following criteria: upstream was 5–25 kbp 5′ of the transcription start site, promoter was 0–5 kbp upstream of the transcription start site, body was between the transcription start site and end, TSE was 0–5 kbp downstream of the transcriptional end, downstream was 5–25 kbp downstream of the transcriptional end, and distant peaks were those not allocated to a gene. Note that the majority of peaks (64%) localized to gene promoters. **(C)** ChIP of five candidate peaks identified by ChIP-Se1. FLAG antibodies were used and ChIP-Seq (EIF1, c-REL, SLC39A13, SMYD5, and ZP3) showed specific MIXL1 binding to both 1MIXL and 2MIXL clones by ChIP normal mouse IgG served as control. Error bars represent standard deviation between triplicates. **(D)** The most common motif in the ChIP-seq peaks are Zinc-Finger binding sites. Motif1 and Motif2 were the two most statistically significant generated using the Multiple EM for Motif Elicitation [MEME] against the peak regions identified in the ChIP-seq analysis. Both motifs are C/G-heavy regions with similarity to known zinc finger motifs.

By taking advantage of the Flag epitope tag and the characterization of two distinct isogenic lines (1MIXL and 2MIXL), we performed a rigorous, high-throughput chromatin immunoprecipitation coupled sequencing (ChIP-Seq) analysis. MIXL1–bound DNA fragments were immunoprecipitated from each clonal line using monoclonal antibodies against Flag epitope. For controls, two separate immunoprecipitations were performed in the U937 vector–transduced cells using (i) FLAG antibodies and (ii) mouse immunoglobulin G (IgG). The sequenced DNA fragments were aligned to the human genome and analyzed in different combinations: 1MIXL-Flag normalized to control-Flag and control-IgG, 2MIXL-Flag normalized to control-Flag and control-IgG, and 1MIXL-Flag and 2MIXL-Flag combined and then normalized to control-Flag and control-IgG. A total of 179 peaks shared by the three groups were examined in further studies (Fig. 2A and Supp. Table S1).

When each of the 179 peaks was localized to its nearest gene locus, 64% of the peaks mapped to gene promoter regions (within 5 kbp upstream of the transcription start site), 8% localized to transcribed regions, 4% were 5–25 kbp upstream of transcription start sites, 1% were within 5 kbp of the 3′ transcription end, and 3% were further downstream, 5–25 kbp from the polyadenylation signal (Fig. 2B). Interestingly, 20% of the peaks (denoted as “distant” in Fig. 2B) were classified as farther than 25 kbp from known genes. To further confirm the global ChIP results, we tested five of the target loci identified (*EIF1*, *c-REL*, *SLC39A13*, *SMYD5*, and *ZP3*) by direct ChIP with Flag-antibody on the three cell lines.(Fig. 2C). All five loci showed specific enrichment, in contrast to the vector transduced U937 cells, confirming the global ChIP-Seq findings.

MIXL1 binding loci were functionally annotated using the gene ontology and tissue expression analyses with the software DAVID (The Database for Annotation, Visualization and Integrated Discovery version 6.7) [31–33]. As anticipated for a homeobox transcription factor MIXL1 occupied genes were involved in broad categories of cellular processes, translation factor activity, nucleic acid binding, organelle, cell part, cell and organelle part functions (Table I and Supp. Table S2). The most significant was the cellular processes class (*P* value = 9.59E-04). Of note, was a tenfold enrichment for factors regulating translation including *EIF1* (NM_005801) confirmed by direct examination (Fig. 2C).

**Table 1:** Gene Ontology of MIXL1 Peaks Identified by ChIP Seq

GO	Term	Count	*P* Value	Fold Enrichment	Benjamini
GO:0009987	cellular process	66	9.59E-04	1.21	0.016
GO:0008135	translation factor activity, nucleic acid binding	5	0.0013	10.36	0.037
GO:0043226	organelle	57	0.0039	1.278	0.042
GO:0044464	cell part	79	0.0040	1.07	0.022
GO:0005623	cell	79	0.0041	1.07	0.015
GO:0044422	organelle part	31	0.0154	1.47	0.042

Based on the structure of homeodomain MIXL1 was predicted to bind a 11 bp motif cooperatively as a dimer [34, 35]. Consistent with this prediction, TAAT motif with a 3 bp spacer i.e TAATTARATTA, was identified by in vitro size selection and confirmed to regulate expression of *Gsc* [36]. *Likewise, Flk1*, and *Pdgfrα* were identified to be activated by a similar motif in mouse embryonic stem cells [37]. To determine whether this motif was enriched in the set of 179 peaks (Supp. Table S1), we analyzed two variants of the motif and a comparable randomized sequence using the Motif Alignment and Search Tool (MAST) (Supp. Fig. S1). Surprisingly, neither motif was more frequent in the 179-peak set than the random sequence. We next performed Multiple Expectation Maximization for Motif Elicitation (MEME) [38] for the sequences within 200 bp of each peak summit. As shown in Fig. 2D, the two highest peaks were:

NC(A/G)(C/G)(C/T/A)AG(G/A)(G/T/A)(G/A)(G/T)(C/A)(G/A)(C/G)(C/T) (width = 15 nucleotides, sites = 65, E-value = 1.1^−027^) and (G/A/T)(A/G/C)(G/A)(G/C/A)(A/C/G)(G/A/T)(G/A)(A/G)(A/G)(G/A/C)N(G/A)(G/A/C)(A/G/T)(G/A) (width = 15 nucleotides, sites = 112, E-value = 3.6^−015^). Interestingly, both these sites harbored a potential zinc finger binding motif consensus. Thus, in the U937 myelomonocytic leukemia system, MIXL1 appears to regulate transcription through either a novel motif or interaction with another DNA-binding protein.

**Figure 3:**
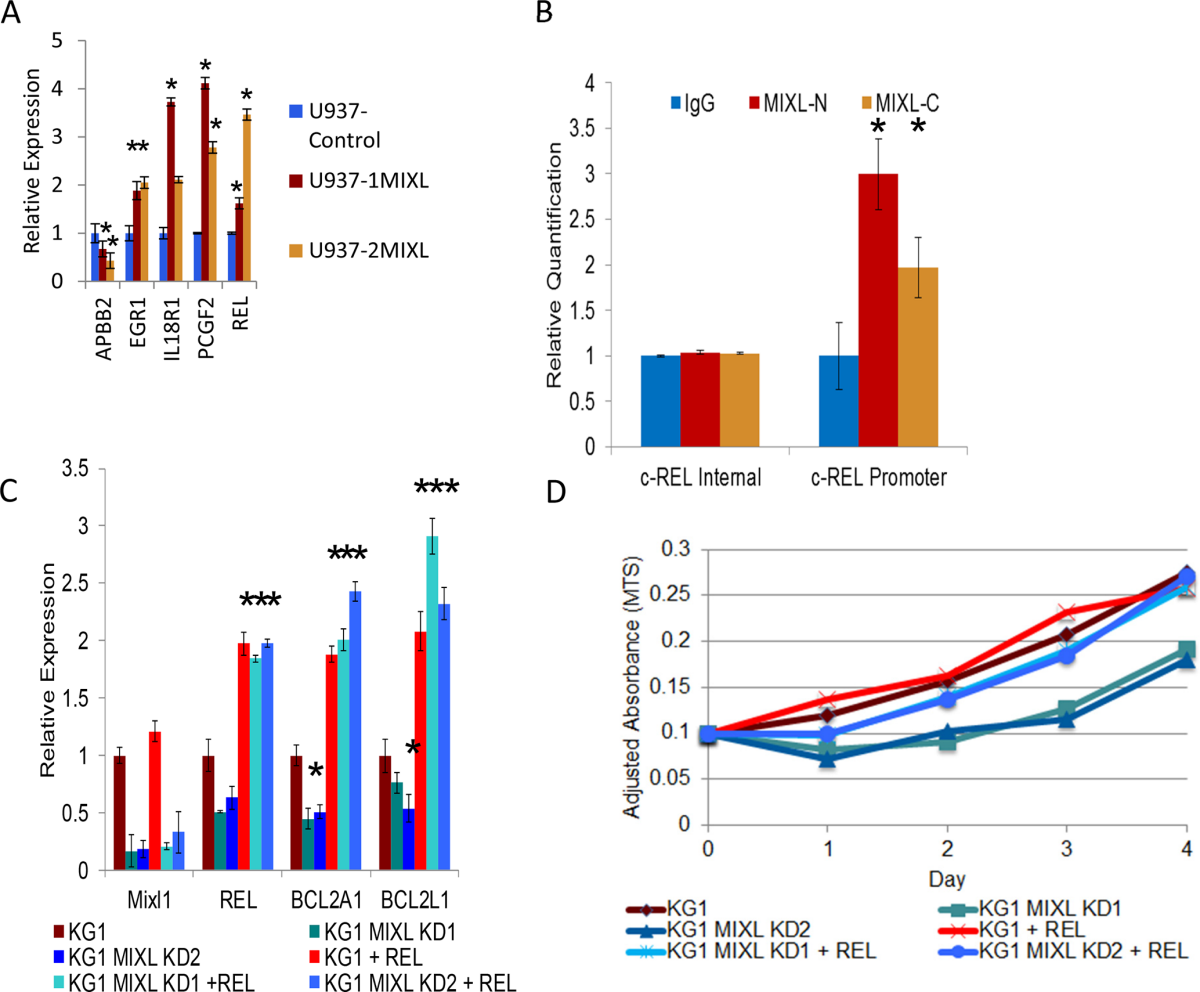
*MIXL1* up regulates *c-REL* expression to enhance anti apoptotic gene transcription **(A)** *MIXL1*-expressing clones show enhanced transcript levels for *c-REL, BCL2A1, and BCL2L1*. Quantitative RT-PCR results show the differences in *c-REL, BCL2A1*, and *BCL2L1* expression levels between the U937 control, 1MIXL, and 2MIXL cells. Expression was normalized to 18S rRNA transcript levels. Error bars represent standard deviation between triplicates. *p < 0.05. **(B)** ChIP localizes endogenous MIXL1 to *c-REL* promoter in KG1 cells. Quantitative genomic PCR analysis shows specific enrichment of endogenous MIXL1 immunoprecipitated with either N-terminal or C-terminal MIXL1 antibodies on the *c-REL* promoter whereas an internal locus within the *c-REL* gene showed no MIXL1 occupancy. Error bars represent standard deviation between triplicates. **(C)** Knockdown of *MIXL1* decreased while enforced expression of *c-REL* increased *c-REL, BCL2A1*, and *BCL2L1* transcript levels. *MIXL1* shRNA lentivirus and *c-REL* retrovirus were transduced into KG1 cells. RT-qPCR was performed in triplicate on RNAs isolated 48 hours after transduction. Expression was normalized to 18S rRNA levels, and error bars represent standard deviation between triplicates. **(D)** *c-REL* over-expression rescues *MIXL1* knockdown–mediated growth arrest in KG1 cells. Growth was measured by MTS assay every 24 hours over a 4-day period in KG1 cells transduced with *MIXL1* shRNA lentivirus and *c-REL* retrovirus. Absorbance was normalized to that of a non-transfected control sample. *p < 0.05.

To further characterize direct transcriptional targets of MIXL1, we performed whole-genome expression analysis using the control and 1MIXL lines on an Affymetrix HG-133 Plus 2.0 microarray (Supp. Table S3). A few differentially expressed genes (*APBB2*, *EGR1*, *IL18R1*, *PCGF2*, and *c-REL*) were validated by reverse transcription coupled quantitative polymerase chain reaction (qPCR) to confirm the global expression profiling results (Fig. 3A). When the ChIP-Seq results were integrated with the global expression profiling results, 82 of the 179 genes identified by ChIP-Seq were either up regulated or down regulated. Sixty-seven of these 82 MIXL1-binding genes were in gene promoters, four were upstream of transcription start sites, nine were in the gene body, and two were downstream of the 3′ transcription end (Supp. Table S4 and S5). Among the genes with expression alterations, the proto-oncogene *c-REL*, cellular homolog of the chicken retroviral oncogene *v-rel* (for reticuloendotheliosis) and a member of the NF-κB family, was of particular interest because of its established role in inducing anti apoptotic genes and the observed decrease in drug sensitivity in MIXL1 expressing clones shown in Fig. 1B [39]. To confirm that endogenous MIXL1 regulated *c-REL* expression, the AML cell line KG1 was tested for occupancy with antibodies against different epitopes (amino and carboxy terminals) on the MIXL1 protein. Fig. 3B confirms localization of MIXL1 to endogenous *c-REL* promoter in KG1 cells. Next, we evaluated the expression of *c-REL* and its transcriptional targets *BCL2A1* and *BCL2L1* (Fig. 3C). As anticipated, KG1 cells expressed *c-REL*, *BCL2A1* and *BCL2L1* transcripts. To determine whether genetic ablation of *MIXL1* affected the expression of its downstream targets, we used two distinct short hairpin RNA (shRNA) lentiviral vectors. *MIXL1* expression was decreased under the knockdown conditions with the two shRNAs but not with the scrambled control (Fig. 3C). Expression of *c-REL*, *BCL2A1*, and *BCL2L1* were all lower under conditions of *MIXL1* knockdown. Forced expression of *c-REL* in *MIXL1* shRNA–expressing cells rescued *BCL2A1* and *BCL2L1* expression, confirming these genes to be transcriptional targets of *c-REL*. We next examined how genetic ablation of endogenous *MIXL1* affected the growth kinetics of KG1 cells (Fig. 3D). While the control shRNA–transfected cells grew exponentially over 4 days as anticipated, the cells with *MIXL1* knockdown showed a diminished doubling time for the first 48 hours. Enforced expression of *c-REL* rescued the retarded growth establishing that loss of *c-REL* expression in the absence of *MIXL1* mediated the diminished growth rate. These results demonstrate *c-REL* to be a direct transcriptional target of MIXL1 in AML cells.

**Figure 4:**
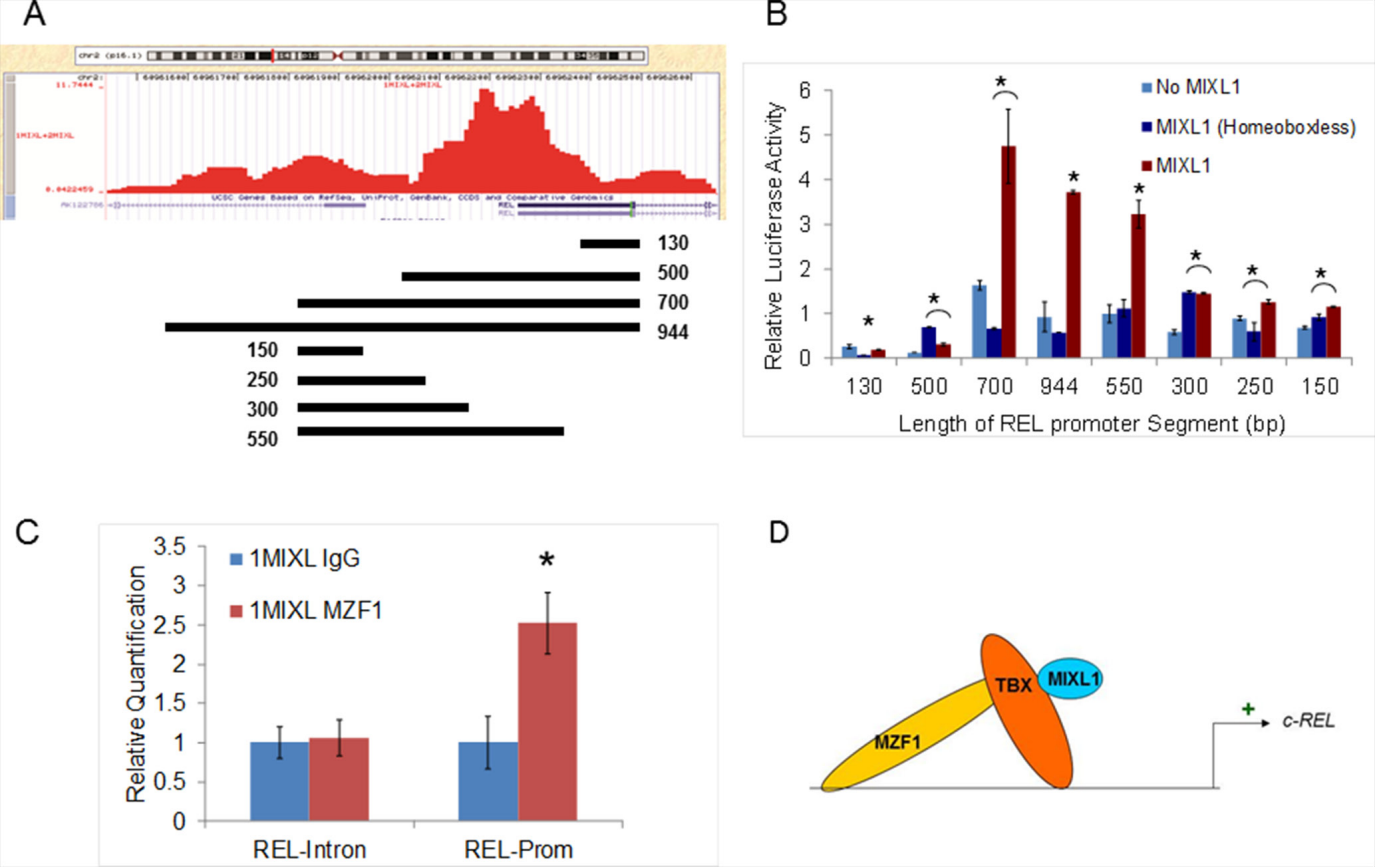
MIXL1 binds to the *c-REL* promoter **(A)** *c-REL* promoter peak region identified by ChIP-Seq, as generated by the University of California, Santa Cruz, genome browser is shown. The location and size of each promoter fragment used for the luciferase reporter assay is displayed underneath. **(B)** MIXL1 binds to a 550-bp region within the *c-REL* promoter. Regions of the DNA depicted in 4A were cloned into the reporter vector pBV-Luc luciferase, which were then transiently co-transfected into HEK293T cells with *MIXL1, MIXL1 Homeobox-less*, or empty expression vector. Equal amount of *Renilla* luciferase co-transfected with the reporter constructs allowed normalization. Luciferase activity of each combination was tested in triplicate after 48 hours. Error bars represent standard deviation between triplicates. **(C)** MZF1 binds to the same locus as MIXL1 on *c-REL* promoter. Quantitative PCR analysis of the identified *c-REL* promoter region and *c-REL* intron control region compared the abundance of each genomic locus immunoprecipitated by either IgG or MZF1 antibodies, normalized to a standard curve. Error bars represent standard deviation between triplicates. *p < 0.05. **(D)** Model depicting a potential MIXL1-TBX-MZF1 multiprotein complex activating *c-REL* transcription.

To further define the promoter elements ChIP peak in the *c-REL* promoter was examined by luciferase reporter assays with nested fragments (Fig. 4A). The reporter constructs were co-transfected with a *MIXL1* expression construct, a HOX-less *MIXL1* expression vector, or an empty vector in HEK293T cells and assayed for luciferase activity. Of these, only the 700-bp and 944-bp promoters were significantly induced by full-length MIXL1 (Fig. 4B). To narrow down the region further, the 5′ part of the 700-bp region was reduced to progressively shorter fragments (Fig. 4A). Among these, only the 550-bp promoter segment was significantly induced by full-length MIXL1 (Fig. 4B).

A search of the 550-bp region for known transcription factor binding motifs identified two NF-κB motifs, a RUNX1 motif, two Sp1 motifs, and four MZF1 motifs (Supp. Fig. S2). MZF1 is a zinc finger transcription factor associated with the myeloid lineage [40]. As the consensus motif identified in the global ChIP was a zinc finger binding motif, qPCR analysis was performed with *c-REL* promoter and control intron primer sets in MZF1-immunoprecipitated 1MIXL cells (Fig. 4C). The promoter region was enriched approximately 2.5-fold in the MZF1-immunoprecipitated fraction, whereas the intronic control region was not. These findings are consistent with *c-REL* promoter activation by MZF1 through direct or indirect interaction with MIXL1 (Fig. 4D).

### Mutually exclusive expression of *MIXL1* and *HOXA9* in primary AML samples

**Figure 5:**
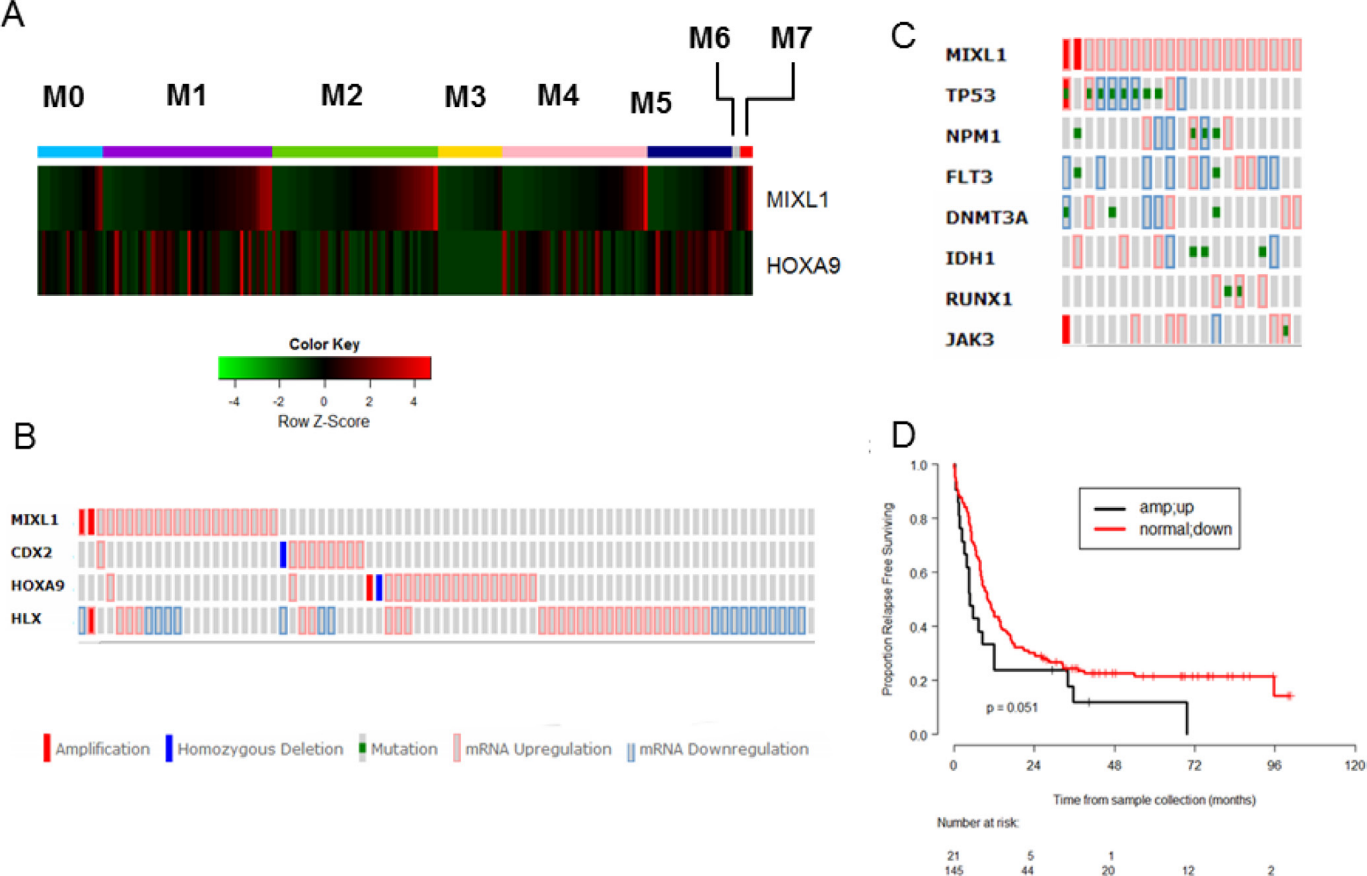
High *MIXL1* expression denotes a distinct subset of AML **(A)** Mutually exclusive expression of MIXL1 and HOXA9 in distinct FAB subsets. The RNA-seq data are publicly available from the TCGA website (https://tcga-data.nci.nih.gov/tcga/). 177 samples of Acute Myeloid Leukemia classified by leukemia French American British morphology code (FAB) and a total of 20319 genes with expression values in the RPKM format were included. The data were quantile normalized using the normalize Quantiles function from the limma package. The expression levels of MIXL1 and HOXA9 of the 177 samples from 8 FAB categories were plotted in the heatmap using the heatmap.2 function in the gplots package of R 3.1.1. Within each FAB category, the samples were ordered according to the expression value of the MIXL1 gene. **(B)** *MIXL1* upregulation identifies a non-overlapping AML subset from those expressing *CDX2, HOXA9*, or *HLX*. TCGA AML patient dataset was queried for alterations in expression as determined by RNA-Seq across 166 AML cases through the cBioPortal database. Each column represents a case of AML. *MIXL1* is amplified or upregulated in 13% of the total AML cases. Note the predominantly non-overlapping expression patterns of *MIXL1, CDX2, HOXA9*, and *HLX*. **(C)** Seventy-six percent (16/21) of *MIXL1*-expressing cases in TCGA AML dataset harbored somatic mutations common in AML (*NPM1, FLT3, DNMT3A, IDH1, RUNX1 JAK3*, and *TP53*). Each column represents a case. **(D)** Relapse-free survival of *MIXL1*-expressing cases is lower than that of non–*MIXL1*-expressing cases. TCGA AML cases were separated into two groups: *MIXL1*-expressing (increase in expression or amplified) and non–*MIXL1*-expressing. Relapse-free survival was then compared between the two groups using the Kaplan-Meier method.

Next we interrogated the significance of *MIXL1* expression in primary human AML. To determine if *MIXL1* expression levels were restricted to specific French American British (FAB) categories of AML, the RNA seq data from TCGA samples were correlated with the 8 FAB subsets. Within each FAB category, the samples were ordered according to the expression levels of *MIXL1* transcripts (Fig. 5A). *MIXL1* expression was seen in a subset of M0, M1, M2, M4 and M7 samples but excluded from M3 and M6. Furthermore, global expression profiling in the 1MIXL1 clonal U937 cells suggested a decrease in *HOXA* cluster transcripts upon *MIXL1* expression. Although *HOXA9* may not be a direct target of repression, because of the established role of *HOXA9* in AML [41, 42], we examined whether the inverse relationship between *HOXA9* and *MIXL1* was also seen in primary AML samples. The FAB based analyses confirmed mutually exclusive expression of *MIXL1* and *HOXA9* within each FAB subset (Fig. 5A). To further refine the identity of *MIXL1* expressing AML samples, we accessed the RNA-Seq data through c-Bioportal [43] which allowed the threshold to be set at a z-score >= 1 for expression. Thus we identified samples with greater than one standard deviation above the mean expression value. By this criterion, *MIXL1* was upregulated in 11.8% of cases and amplified and overexpressed in 1.2% of cases (Fig. 5B). *HOXA9* was up regulated in 10.8% of cases. Once again, very few samples had expression in either gene suggesting a mutually exclusive expression pattern. Furthermore, higher transcript levels of the three HOX genes previously characterized to be important in AML (non-clustered HOX genes *CDX2 [10], HLX [12], HOXA9*) and *MIXL1* were mutually exclusive with a few exceptions (Fig. 5B). To rule out the apparent lack of overlap was due to the low threshold used, we validated this method by examining the transcript levels of *PBX3, MEIS1, HOXA9* encoding members of a multi protein complex [44–46]. Concomitant over expression of all three members in this AML dataset confirmed the applicability of this method (Supp. Fig. S3A).

To determine whether *MIXL1* expression was associated with any of the commonly identified somatic mutations the TCGA samples were queried for *NPM1, FLT3, DNMT3A*, and *TP53* mutations (Fig. 5C). The most frequent of these alterations were *TP53* mutations, seen in eight (38%) of the cases with *MIXL1* expression; interestingly, the eight cases constituted 66% of the 12 AML cases with *TP53* mutations in this dataset. The other recurrent mutations were less frequent. Four cases had mutations in *NPM1*; *DNMT3A, and IDH1* mutations appeared in three cases each; *FLT3* and *RUNX1* mutations appeared in two cases each; and a single case had a *JAK3* mutation. In total, 76% (16/21) of the cases with high *MIXL1* expression had common AML associated mutations. To validate this approach we queried the mutational profile of *HOXA9* over expressing samples; these were predominant in the *NPM1* mutated cases (Supp. Fig. S3B) consistent with pediatric AML studies and murine models of *NPM1* mutations [47, 48].

Finally, to determine whether *MIXL1* expression impacts outcome in AML, the relapse-free survival rates were compared between samples with expressing MIXL1 and those without readily detectable expression. The cases expressing *MIXL1* had notably shorter relapse-free survival than those without high *MIXL1* ( *p* = 0.051; Fig. 5D). In contrast, samples with increased expression of *CDX2*, *HLX* and *HOXA9* did not attain statistically significant event free survival by these criteria (Supp. Fig. S4 A-C).

### BMP4 induces *MIXL1* in HSPCs and *MIXL1*-expressing AML cells are sensitive to the BMP inhibitor LDN-193189

**Figure 6:**
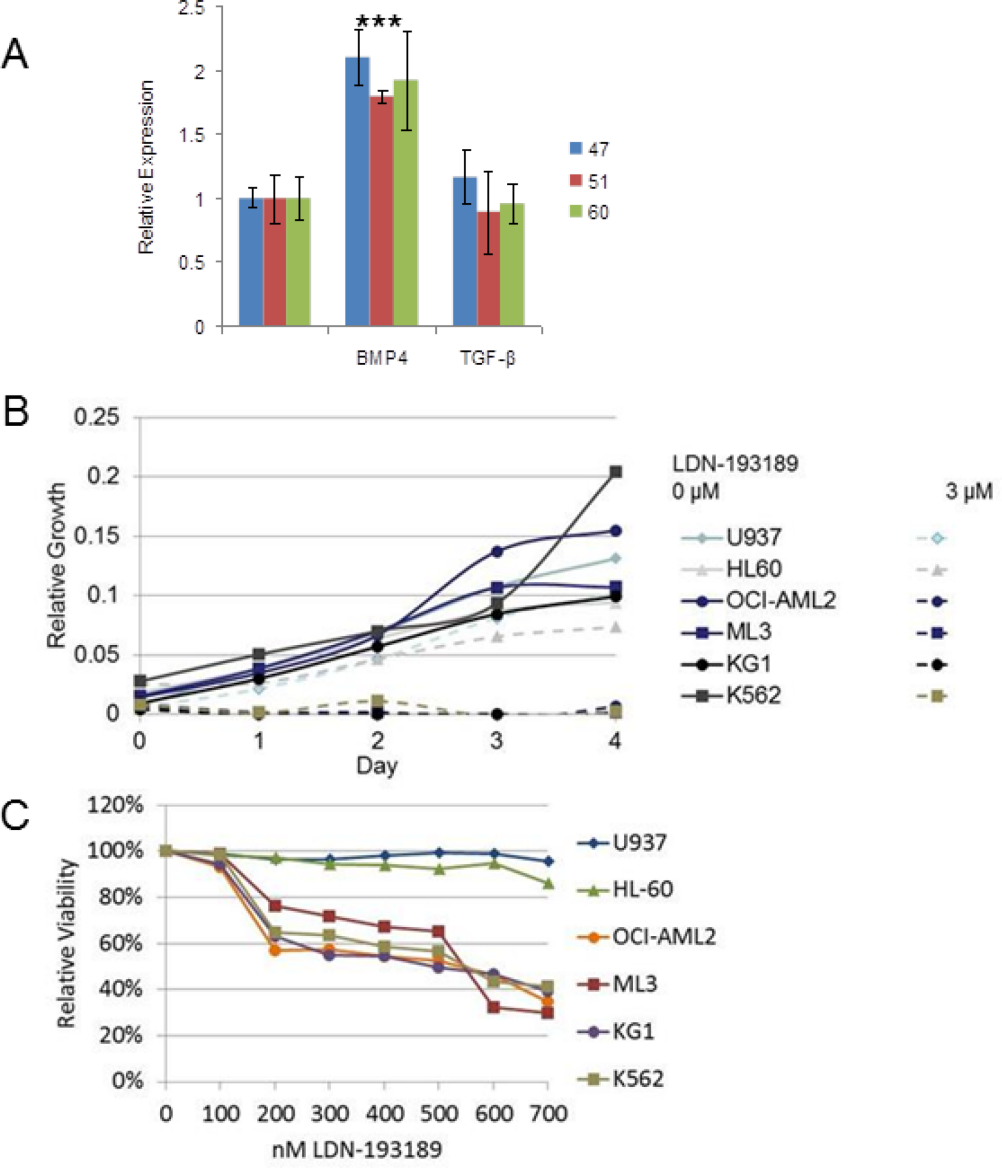
BMP4 induced *MIXL1*, an important survival axis and therapeutic target in AML **(A)** MIXL1 expression increased 2-fold in CD34^+^ HSPCs treated with BMP4. CD34^+^ HSPCs from three cord blood donors (unique donor number 47, 51, 60) were cultured with either 50 ng/ml BMP4 or 2 ng/ml TGF-β1 for 2 hours. *MIXL1* transcript levels were quantified by RT-qPCR using 18S rRNA as normalization control. Error bars represent standard deviation between triplicates. *p < 0.05. **(B)** LDN-193189 at 3 μM was cytotoxic to OCI-AML2, ML3, KG1, and K562 cells but not U937 and HL60 cells. Each cell line was treated with vehicle or 3 μM LDN-193189 on day 0, and viability was measured every 24 hours by MTS assay in triplicate. **(C)** OCI-AML2, ML3, KG1, and K562 were sensitive to 200 nM LDN-193189, while the non–MIXL1-expressing lines U937 and HL60 were unaffected. Each cell line was treated in triplicate with 0–700 nM LDN-193189, with the drug or control medium replenished every 24 hours, for 4 days. Viability on day 4 was assayed by MTS assay. Absorbance was normalized to that of control samples treated with vehicle only.

To determine whether there is a ligand preference between BMP and TGF-β for *MIXL1* induction human cord blood–derived HSPCs from three donors were short-term cultured in the presence of BMP4 or TGF-β. Upon 2 hours of treatment with BMP4, *MIXL1* transcript levels were 1.8–2 times higher than control HSPCs, whereas *MIXL1* was not induced by TGF-β (Fig. 6A). These results suggested that BMP4 is a preferred ligand upstream of *MIXL1* in HSPCs. Since the HSPCs used are an enriched lineage-negative population composed primarily of progenitors with rare (<10%) stem cells, the response to BMP4 was likely in the progenitors. Notably, the consistent induction of *MIXL1* in three donor-derived HSPC lines signified a fundamental difference between the BMP4 and TGF-β responses in these cells.

*MIXL1* induction by BMP4 raised the possibility that AML cells that express *MIXL1* have increased sensitivity to BMP pathway inhibition. The ALK2/3/6 (ACVR1, BMPR1A, and BMPR1B) inhibitor LDN-193189 shows a preference for BMP and activin signaling over TGF-β signaling [49]. Four of the previously characterized high *MIXL1*–expressing cell lines (OCI-AML2, KG1, ML3, and K562) and two cell lines lacking *MIXL1* expression (U937 and HL60) were grown in medium containing 3 μM LDN-193189 for 4 days. LDN-193189 was strongly cytotoxic to all the *MIXL1*-expressing cell lines, whereas HL60 and U937 cells recovered by day 4 after an initial setback (Fig. 6B). Since the high dose of LDN-193189 could have had off-target effects [50], we performed a dose-response assessment in the 0–700 nM range (Fig. 6C). Once again, the *MIXL1-*negative cell lines U937 and HL60 were relatively unaffected, whereas the *200–700nM range was cytotoxic to MIXL1*-expressing cells. The differential sensitivity of these AML cell lines to LDN-193189 together with the BMP4-induced expression of *MIXL1* suggests that inhibition of the BMP receptor kinase may be an effective therapeutic approach for *MIXL1* expressing AML cells. Furthermore, to exclude potential off target effects specific to MIXL1 expressing cells, we tested LDN-193189 response in the stably transfected clonal U937 cells, 1MIXL1. There was no significant difference in sensitivity to LDN-193189 over 1-3 uM range between control U937 and 1MIXL1 cells suggesting the cytotoxicity seen in AML cell lines to be primarily through type I BMP receptor pathway (Supp. Fig. S5).

## DISCUSSION

In this study, we identify *c-REL* as a direct transcriptional target of *MIXL1* and BMP4 as a ligand upstream of *MIXL1*. These findings implicate the BMP4–*MIXL1*–*c-REL* axis in AML pathogenesis. Notably, this pathway may be therapeutically targeted with type I BMP receptor kinase inhibitors.

### Transcriptional targets of MIXL1

Several novel transcriptional targets of MIXL1 were identified by ChIP-Seq and the importance of a number of these molecules and relevant pathways will be examined in the future. For this initial report, *c-REL* was of immediate relevance because of its established role in the activation of the anti-apoptotic gene *BCL2L1* encoding Bcl-XL protein [51]. Although the NFκB pathway is canonical, recent evidence suggests potential functional variations in the activity of *c-REL* depending on its subunit composition [52]. Thus, c-*REL* homodimers or heterodimers with NFκB2 or RELB may have varied transcriptional responses [53]. Among these *BCL2L1* encoding the BCL-X_L_ protein is critical as silencing this pathway synergizes with hypomethylating agents in AML [54]. Likewise, retroviral expression of *BCL2A1* a lesser characterized anti-apototic molecule, enhances engraftment potential of hematopoietic stem cells and subsequent transformation to transplantable malignancies [55].

Although intriguing initially, recent studies suggest the possibility of a potential MIXL1-Tbox-MZF1–containing multiprotein complex. First, our unbiased target motif search based on rigorous ChIP sequencing studies identified potential zinc finger binding sites (Fig. 2D). Second, MIXL1 and the T box factors T, Eomes, Tbx6, and Tbx20 interact directly in embryonic stem cells to regulate transcription [56]. Third, TBX20-MZF1 interaction has been identified in a high-throughput mammalian transcription factor interaction screen [57]. Therefore, our findings raise the possibility with a MIXL1/Tbox/MZF1 multiprotein complex that mediates transcriptional regulation of *c-REL*. Clearly, further studies are necessary to identify T box factor that bridges MIXL1 and MZF1.

Thus, the absence of the consensus TAAT motifs in the ChIP targets together with MZF1 localization to the *c-REL* promoter (Fig. 4C) suggest that MIXL1-MZF1–containing multiprotein complexes, rather than MIXL1 homodimers, may be important in AML. Such a scenario would be analogous to, the paired type homeo box factor NKX2-5, TBX3, and zinc finger factor GATA4 multiprotein complex that plays a central role in cardiac development [58].

### *MIXL1* as a novel marker in AML

*MIXL1*-expression makes up 13% of AMLs in the TCGA dataset, a group both distinct from and larger than the *CDX2*- and *HOXA9*-expressing subsets (Fig. 5A and 5B). Using the cBioPortal RNA-Seq data, we set the threshold low so that subtle yet pathologically relevant alterations may be detected. This group had a slightly lower rate of survival than those not expressing *MIXL1* (Fig. 5D), suggesting that *MIXL1* may be an independent prognostic marker.

Our analysis of TCGA AML dataset although small, agrees overall with the larger global profiling results for HOX expression in AML: *FLT3*-mutated AMLs have higher *HOXA9* expression [59], AMLs with mutant *NPM1* also show aberrant *HOXA9* expression [47], and the coordinate expression of three established members of a transcriptional complex—*HOXA9*, *MEIS1*, and *PBX3*—is common (Supp. Fig. 3A). Notably, the search for *MIXL1* expression in TCGA AML cases uncovered an elegant stratification of HOX expression, including some patterns of mutual exclusion. The mutual exclusivity of MIXL1 and HOXA9 expression (Fig. 5A and 5B) suggests the specific homeobox genes might cooperate with distinct driver mutations. Lack of MIXL1 expression in AML M3 acute promyelocytic leukemia (Fig. 5A), frequently associated with the PML-RARA translocation arising in the context of myeloid restricted gene expression program is also consistent with such a model. Collectively, these results suggest that quantitative evaluation of both clustered and non-clustered HOX transcripts in AML may stratify AML further. Such an evaluation may elucidate whether HOX expression reflects the stage of progenitor maturation which cooperates with the driver mutations. Interestingly, a recent report in pediatric AML suggests a similar pattern of mutual exclusion; namely *HOXA* and *HOXB* down regulation in cases of RUNX1 or *PML/RARA* translocation or *CEBPA* double mutations, *HOXA* up regulation, *HOXB* down regulation in cases of MLL translocation and *MYST* up regulation, or *HOXA* and *HOXB* upregulation in *patients with NPM1* mutation, and *NUP98* translocations. Thus the HOX expression pattern could be segregated by distinct cytogenetic or driver mutations [60, 61].

Functionally, the decreased drug sensitivity of MIXL1 expressing U937 clones shown in Fig. 1A is suggestive of an antiapoptotic advantage conferred through *c-REL.* Whereas this is the only readily detectable difference in U937 cells, in KG1 cells genetic ablation of endogenous MIXL1 results in loss of viability (Fig. 3D) which is rescued by *c-REL.* Such a significant difference between the two AML cell lines could be explained in part by the fundamental difference in the driver mutations in these cell lines. U937 cells harbor a CALM-AF10 translocation [62] whereas KG1 is driven by a constitutively activated FGFR1 tyrosine kinase due to FGFR1OP2-FGFR1 translocation [63]. CALM-AF10 fusion induces HOXA over expression in murine models [64]. Recent studies demonstrate CALM-AF10 mediated epigenetic reprogramming of HOXA locus to be dependent on nuclear exporter CRM1. Thus the transcriptional circuit in U937 cells is distinct from that of KG1. Therefore, U937 cells may be addicted to other HOX cluster mediated growth and clonogenic advantage whereas KG1 cells may be dependent on MIXL1.

### Therapeutic potential of targeting type 1 BMP receptor

**Figure 7:**
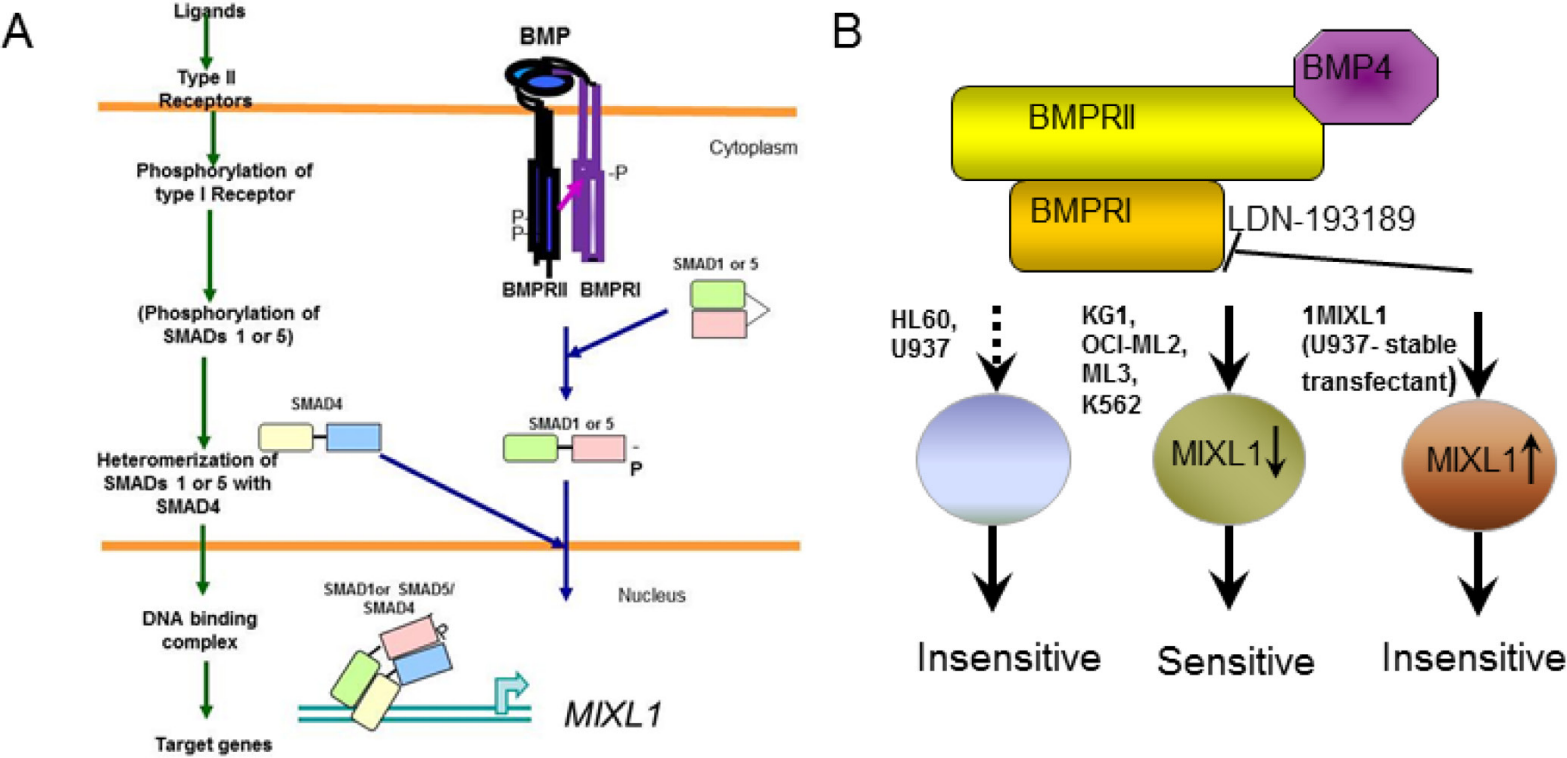
BMP4 induced *MIXL1* an important survival axis and therapeutic target in AML **(A)** Canonical BMP signaling pathway. Upon BMP binding to type II receptor (BMPRII), type I receptor BMPRI is phosphorylated. Activated BMPRI phosphorylates SMAD1 or 5 which heterodimerizes with SMAD4 to up regulate MIXL1 expression. **(B)** BMPR1 mediated signal is hypothesized to induce endogenous MIXL1 expression in KG1, OCI-ML2, ML3 and K562 cell lines. These cells are sensitive to BMPR1 inhibitor LDN-193189. Enforced expression of MIXL1 in U937 cells 1MIXL1 is independent of BMP signaling and therefore insensitive to LDN-193189. In HL60 and U937 cells which are insensitive to LDN-193189, the pathway may be absent or modified.

*Mix.1* is induced in Xenopus embryos by BMP4 or activin A in a SMAD5-dependent manner [22, 23], and *MIXL1* can be induced by TGF-β in human hepatocellular carcinoma [65] and in mouse embryonic stem cells [26]. TGF-β from the glial cells within the bone marrow niche is thought to maintain hematopoietic stem cell quiescence [27]. BMP proteins regulate maintenance, proliferation, and repopulating activities of hematopoietic progenitors [66–68]. A recent study reported that BMP receptor IB is required for the expansion of primitive chronic myelogenous leukemia stem cells, raising the possibility of targeting this pathway in chronic myelogenous leukemia [69]. Additionally, a subset of pediatric AML cases with a novel translocation *CBFA2T3-GLIS2* and poor outcomes express BMP2 constitutively and respond *in vitro* to dorsomorphin, a precursor molecule to LDN-193189. Importantly, these studies demonstrated murine bone marrow cells transduced with *CBFA2T3-GLIS2* to be more sensitive to dorsomorphin than wild type cells in colony forming assays [70]. Our findings demonstrate for the first time that *MIXL1* is induced in human HSPCs by BMP4. Future studies will uncover whether the BMP ligand for *MIXL1* induction in HSPCs is stromally derived or autocrine. Above all, our findings suggest the ACVR1/BMPR1 pathway to be preferentially engaged to induce *MIXL1* in hematopoietic stem cells or progenitors. As shown in Fig. 7A the canonical type II receptor dimer phosphorylates the type I receptor upon ligand binding [25]. The phosphorylated type I receptor in turn may phosphorylate SMADs 1 or 5 which translocate to the nucleus to activate MIXL1 expression. Our findings are consistent with existence of such a pathway in MIXL1 expressing KG1, OCI-ML2 and K562 cells. Of note, studies from the Zon laboratory showed BMP2 treatment of K562 and U937 recruited phosphor SMAD1 with lineage specific factors (erythroid factor GATA1 in K562 cells derived from chronic myelogenous leukemia in erythroid blast crisis and CEBPA in U937 myelomonocytic cells) [71]. A search of the ChIP seq data from this study in the public domain revealed SMAD1 binding to MIXL1 promoter in K562 and not U937 cells. Thus a potential model shown in Fig. 7B suggests expression of MIXL1 induced by BMP or related ligand can be inhibited by LDN-193189 to induce cell death. Recent studies in lung cancer cell lines demonstrate potent inhibition of clonogenic potential of lung cancer cells by LDN-193189 in a SMAD dependent manner [72].

Of note, two thirds of TCGA AML samples with *TP53* mutations, a subset with very few therapeutic options, had high *MIXL1* expression. The significance of *MIXL1* expression is further heightened in the context of BMP pathway suppression in the AML cell lines KG1 and K562, which have compromised TP53 activity. The differential cytotoxicity by 3 μM LDN-193189 specific to those lines indicate that a defined subtype of AML may be responsive to type I BMP receptor kinase suppression. LDN-193189 also inhibits non canonical BMP signaling [73]. This would be a significant boon, as LDN-193189 has already been used in rodent models of hepcidin-induced chronic anemia and fibrodysplasia ossificans progressiva (FOP) with constitutive activation of ACVR1, a type 1 BMP receptor kinase [49, 74]. A caveat, however, is that LDN-193189 inhibits ALK2/3/6 kinases (ACVR1, BMPR1A, and BMPR1B) [75], and its activity may inhibit other type I receptors of the TGF family ligands. Other kinases that LDN-193189 can bind and inhibit include SIK1, ABL, VEGFR, YES1, CAMKK2 [76]. While many of these kinases may be useful therapeutic targets themselves, the role that inhibition of BMP or related ligands would play in AML needs further evaluation. Indeed, a soluble activin receptor that sequesters the ligand is showing promise in myelodysplasia, a clonal disorder and common precursor to AML [77, 78].

In summary, we define for the first time the potential role of *MIXL1* in human AML and implicate *c-REL* as a direct transcriptional MIXL1 target to confer an anti apoptotic advantage. More extensive studies are required to define mutually exclusive expression of *MIXL1* from other HOXs, inducibility of MIXL1 by BMP4 and above all preferential sensitivity of MIXL1 expressing AML cells to BMPR1 inhibitors. The findings reported here provide a compelling rationale for future investigations which may lead to a novel targeted therapy for an aggressive subset of AML.

## MATERIALS AND METHODS

### Cell culture

AML cell lines U937, HL60, OCI-AML2, and ML3 and chronic myeloid leukemia cell line K562 were grown in 5% CO_2_, 95% humdified air at 37°C in RPMI 1640 with 10% fetal bovine serum. KG1 cells were grown in RPMI 1640 with 20% fetal bovine serum. Human embryonic cell line HEK-293T was grown in Dulbecco's Modified Eagle Medium with 10% fetal bovine serum.

We established two clonal lines, 1MIXL and 2MIXL, expressing amino FLAG- and HA-tagged MIXL1 driven by a Tet response element in the U937 clonal line expressing the tetracycline-controlled transactivator protein as previously described [28, 79, 80]. A clonal line transfected with the vector served as a control. After multiple passages, we noted that both lines were leaky regardless of the selection pressure. Therefore, 1MIXL and 2MIXL were treated as stable MIXL1 overexpression clones without any selection agent or tetracycline.

### Human cord blood hematopoietic stem progenitors

Human CD34^+^ progenitor cells were acquired from cord blood as described previously [81]. Briefly, mononuclear cells were isolated from umbilical cord blood by density gradient separation, followed by enrichment of CD34^+^ cells via magnetic bead separation. CD34^+^ HSPCs were cultured in TGF-β or BMP4 for 2 hours.

### Lentiviral knockdown

Lentiviral constructs were from Open Biosystems (Pittsburgh, PA) and designated as follows: MIXL1 KD1 = TRCN0000019155, MIXL KD2 = TRCN0000019156, and Rel Expression = ccsbBroad304_11094. The protocol for lentiviral production was similar to that of Moffat et al. in HEK293T cells by transient transfection of the lentiviral construct, the envelope construct pCMV-VSV-G, and the gag-pol construct at a ratio of 2:1:1, respectively [82].

Transductions of AML cells were performed by resuspending 2 × 10^5^ cells in 1 ml of virus-containing medium with 8 μg/ml polybrene and incubating the suspension at 37°C for 24 hours prior to pelleting by centrifugation and resuspension in growth medium. Growth assay and expression experiments were performed 48 hours after transduction in medium containing 2 μg/ml puromycin.

### Chromatin immunoprecipitation

Chromatin immunoprecipitation was based on the procedure used by Chadee et al. [83] with the following modifications. For each U937 cell line, a total of 10^8^ cells were cross-linked with 1% formaldehyde in growth medium at 37°C for 20 minutes. Cells were harvested by centrifugation at 3000 rpm for 10 minutes and resuspended in 500 μl of radio-immunoprecipitation assay (RIPA) lysis buffer. After 10 minutes on ice, the cells were sonicated 20 times at 4–5 watts for 20 seconds, with a rest time of 40 seconds between each sonication. The samples were then centrifuged for 5 minutes at 4°C and precleared with 20 μl of A/G agarose slurry for 1 hour at 4°C. Two aliquots from each lysate were processed as follows: Flag-IP with 240 μl of the lysate, 250 μl of lysis buffer, and 8.4 μg of mouse anti-flag antibody (anti-Flag-M2; Sigma-Aldrich, St. Louis, MO) and IgG-IP with 240 μl of the lysate, 250 μl of lysis buffer, and 8.4 μg of mouse IgG. The samples were incubated overnight at 4°C with rotation and then incubated for an additional hour with 20 μl of A/G agarose slurry. The agarose beads were recovered by centrifugation and washed for 15 minutes each in RIPA lysis buffer, high-salt RIPA buffer, lithium chloride RIPA buffer, and finally TE buffer prior to proteinase K and RNAse treatment at 37°C overnight. The samples were incubated for 6 hours at 65°C to reverse the cross-linking. DNA was precipitated overnight at –20°C in 75% ethanol and then washed twice in 75% ethanol. For each sample, DNA from a 20-μl aliquot of the pre-immunoprecipitation lysate diluted with 180 μl of TE buffer served as another control for target amplification. The DNAs were resuspended in 50 μl of ddH_2_O, and the pre-immunoprecipitation sample was diluted with 450 μl of ddH_2_O. Fifty nanograms each of immunoprecipitated DNAs from (1) U937 control IgG-IP, (2) U937 control Flag-IP, (3) U937 1MIXL Flag-IP, and (4) U937 2MIXL Flag-IP were used to construct libraries at the Sequencing and Microarray Facility at The University of Texas MD Anderson Cancer Center using the Beckman SPRIworks system, Pasadena, CA. Illumina analysis pipeline (GAPipeline-1.5.0 San Diego, CA) was used for base calling and alignment to the human genome. Peak calling was done by MACS v1.3.7.1 with *p* ≤ 1e-5 considered significant. Peaks were identified against the human genome (University of California, Santa Cruz, genome browser assembly hg18, NCBI36) using genome model-based analysis of ChIP-Seq (MACS) [84]. Unique peaks were generated by normalizing to the two control samples in three combinations: Flag-1MIXL to IgG-control and Flag-control, Flag-2MIXL to IgG-control and Flag-control, and Flag-1MIXL and Flag-2MIXL combined to IgG-control and Flag-control. The primary dataset used for analysis comprised the overlapping peaks in all three analyses. The combined dataset was tested for predicted Paired-Q9 binding motifs [34, 35] using MAST [85], and enriched motifs were identified using MEME [38].

ChIP-PCR confirmation was performed by SYBR green system quantitative PCR using an exonic REL region primer set as a control and the following primer sets: REL internal region (5′-TTACCAGGATTTTGGCAAGG-3′ and 5′-CAGGCAGTTTGGGGATAAGA-3′), REL peak (5′-GGAACCACCTCTCGAAAACC-3′ and 5′-TCCAGGTTGTTCTTCCGAGT-3′), EIF1 peak (5′-TGACTCCGTGGGTAGTAGGG-3′ and 5′-CCTTCTTGACCCTGTTGCAT-3′), SLC39A13 peak (5′-CCTGAGGTTCCCAGTGAAAA-3′ and 5′-GAGGACTACTGTGCGCTCCT-3′), SMYD5 peak (5′-TTCCCCCTTTCATGACTCTG-3′ and 5′-CTCAGCTCAGTCCCCAAGAG-3′), and ZP3 peak (5′-ACCTCAGCCTCCCCAGTAGT-3′ and 5′-TTGATCCAAAAGCAGCTGAA-3′). For ChIP-qPCR against endogenous proteins, 5 μg of anti-MIXL1-N, or 5 ug of anti-MIXL1-C antibodies were used for immunoprecipitation.

### Global expression profiling

To identify potential targets of the MIXL1 transcription factor, we performed global expression profiling analysis on *MIXL1*-expressing cells by microarray. The 1MIXL and control cell lines were cultured without Tet for 24 hours at 5 × 10^4^ cells/ml. RNA was extracted from 5 × 10^6^ cells by RNeasy Mini Kit (QIAGEN, Valencia, CA). Extracted RNA was hybridized against a HG-U133A Microarray (Affymetrix, Santa Clara, CA).

For analysis, dChip analyzer software [86] was used, normalizing the 1MIXL dataset to the control dataset, and gene expression models were obtained through the Perfect Model–only approach. Differentially expressed genes were defined as genes in which the difference between the detected expression levels was at least 100 and the ratio was at least 1.2.

### Public access

The ChIP Seq data can be accessed at GSE52781 and the expression profiling is designated GSE52622

### RT-qPCR

RNA was extracted using the RNeasy Mini Kit (QIAGEN), and cDNA was prepared by reverse transcription using SuperScript II (Life Technologies, Carlsbad, CA). The samples were assayed by qPCR in triplicate using the following TaqMan primers: MIXL1 (Hs00968440_m1), REL (Hs00231279_m1), BCL2L1 (Hs99999146_m1), BCL2A1 (Hs00187845_m1).

For CD34^+^ cells, RNA was harvested in triplicate from approximately 2,500 cells using the RNeasy Plus Micro Kit (QIAGEN) 2 hours after the addition of either 50 ng/ml BMP4 (R&D Systems, Minneapolis, MN) or 2 ng/ml TGF-β1 (Sigma-Aldrich) in X-VIVO 15 medium (Lonza, Allendale, NJ), and then 100 ng from each sample was reverse transcribed and assayed.

### Immunoblotting

The Western blotting technique was performed as previously described [17] using the following modifications: Samples lysed with whole cell lysis buffer (20 mM Tris, 250 mM NaCl, 2 mM EDTA, 1% Triton X-100, 1 mM DTT 2 μg/ml aprotinin, 2 μg/ml leupeptin, 2 μg/ml pepstatin A, 1 mM NaVO_3_, 1 mM PMSF), were resolved on 10% NuPAGE gel (Life Technologies), and a mixture of Tris-buffered saline and Tween 20 (50 mM Tris, 150 mM NaCl, and 0.05% Tween 20 adjusted to pH 7.6) was used for immunoblotting. The primary antibodies used were anti-MIXL1-N at a dilution of 1:1,500 and beta-actin (Sigma Aldrich) at 1:5,000. The secondary antibodies used were anti-rabbit horseradish peroxidase (GE Healthcare Wauwatosa, WI at 1:10,000 and anti-mouse horseradish peroxidase (GE Healthcare) at 1:7,000 for the beta-actin primary antibody.

### Luciferase assay

Luciferase constructs were generated by PCR amplification of normal human DNA segments of the REL peak region identified by ChIP-Seq. Promoter segments were amplified from genomic DNA by PCR using the following probes:

Rel-R-EcoR1 (5′-ctgtgaattcCGCAGTCAGTCAGTCAGGAG-3′),
Rel-FM-130 (5′-ctgtacgcgtAGAATTCAGGGGTTGGGAAG-3′),
Rel-FM-500 (5′-ctgtacgcgtGGAAGAACAACCTGGAGGAG-3′),
Rel-FM-700 (5′-ctgtacgcgtGAACCACCTCTCGAAAACC-3′),
Rel-FM-944 (5′-ctgtacgcgtGGAGCTTTGGAGTCAGACAA-3′),
Rel-RE-150 (5′-ctgtgaattcCAGGTTGTTCTTCCGAGT-3′),
Rel-RE-200 (5′-ctgtgaattcGGCTAGCAGCGTGAGAAGG-3′),
Rel-RE-300 (5′-ctgtgaattcGACGCAGCAACCCTCACC-3′), and

Rel-RE-580 (5′-ctgtgaattcAACCCCTGAATTCTTGCAC-3′). Each was then sub-cloned into the pBV-Luc vector between the Mlu1 and EcoR1 sites. 293T cells were transfected with 200 ng of expression vector, 200 ng of luciferase vector, and 0.2 ng of *Renilla* luciferase vector by Lipofectamine (Invitrogen, Grand Island, NY). The activity was then tested by the Dual-Luciferase Reporter Assay System (Promega, Madison, WI) 48 hours post-transfection.

### Methanethiosulfonate (MTS) assay

The MTS assay in which a tetrazolium compound is reduced by cellular NADH to generate colorimtrically detectable formazon product was used to quantify cell number and viability. The amount of product formed was a direct function of the number of viable cells. For comparative analysis of the effects of doxorubicin on U937 *MIXL1*-overexpressing lines, doxorubicin (Sigma-Aldrich) was added to the cell lines at 0–2 μM in triplicate, for 24 hours of incubation before MTS treatment. Comparison of knockdown cell lines was performed by plating 3 × 10^4^ cells/well for each line into a 96-well plate in triplicate and then adding the CellTiter 96 AQueous Non-Radioactive Cell Proliferation Assay kit (Promega) with a 1-hour incubation, following the manufacturer's instructions. For LDN-193189 treatment, each cell line was grown in growth medium containing either 3 μM LDN-193189 (Cellagen Technology, San Diego, CA) or vehicle (dimethyl sulfoxide) in triplicate for 4 days with growth measured every 24 hours. For the dose-response studies, each line was grown in 0–0.7 μM LDN-193189 in triplicate replenished with fresh drug-containing medium every 24 hours to account for loss of drug activity at 37°C, and the MTS assay was performed at day 4.

### Bioinformatics and data mining

The functionality of the gene list from CHIP-seq, was analyzed for gene ontology analysis and tissue expression with the software DAVID (The Database for Annotation, Visualization and Integrated Discovery version 6.7 [31–33]. The functional analysis in DAVID evaluated gene-to-gene similarity by utilizing the terms from gene ontology [87]

The RNA-seq data were publicly available from the TCGA website (https://tcga-data.nci.nih.gov/tcga/). 177 AML samples stratified by leukemia French American British morphology classification (FAB) and a total of 20319 genes with expression values in the RPKM format were included. The data were quantile normalized using the normalizeQuantiles function from the limma package [88] The expression levels of MIXL1 and HOXA9 of the 177 samples from 8 FAB categories were plotted in the heatmap using the heatmap.2 function in the gplots package of R 3.1.1 (Development Core Team. R Foundation for Statistical Computing. Vienna, Austria: 2009. R: A language and environment for statistical computing. ISBN 3-900051-07-0, URL http://www.R-project.org). Within each FAB category, the samples were ordered according to the expression value of the MIXL1 gene. Additionally, TCGA AML database [8] was accessed and analyzed through the cBioPortal [43, 89] for cases with a mutation, copy-number alteration, or expression change over a threshold of 1.0 in the genes of interest. For statistical analysis, clinical data and expression data from the 166 samples were collected from the TCGA AML dataset for whom outcome was available [8] and compared between the amplified or upregulated group and the normal, loss, or down-regulated group for each gene. The unadjusted distribution of relapse-free survival was evaluated by the method of Kaplan and Meier [90], and the differences in the distributions between expression levels were compared using the log-rank test.

Transcription factor binding motifs in the *c-REL* promoter were identified using TFSEARCH [91].

## SUPPLEMENTARY FIGURES AND TABLES








