# Supplementary material for: A role for BMP-induced homeobox gene MIXL1 in acute myelogenous leukemia and identification of type I BMP receptor as a potential target for therapy
Source: Oncotarget. 2014 Dec 17;5(24):12675–93. doi: 10.18632/oncotarget.2564 (PMC4350356; doi:10.18632/oncotarget.2564)
Supplement: Supplementary file 1 [file oncotarget-05-12675-s001.pdf]

SUPPLEMENTARY FIGURES AND TABLES

Motif1

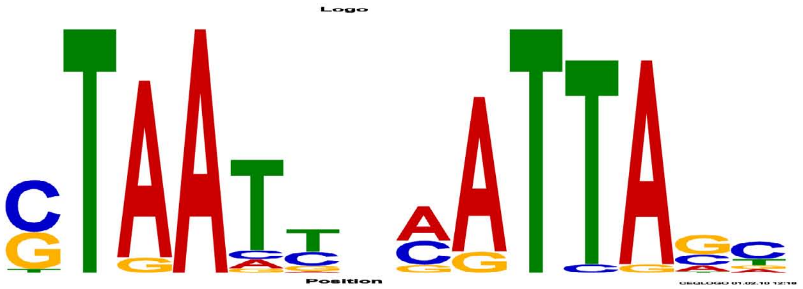

Motif1	(179 peaks)		
pvalue	1e-4	5e-4	1e-3
1000bp	3<br>4.97	31<br>44.6	59<br>77.75
500bp	1<br>1.65	9<br>18.2	18<br>35.65
200bp	0<br>0.43	2<br>5.76	3<br>12.06
100bp	0<br>0.18	0<br>2.75	1<br>5.62

Motif2

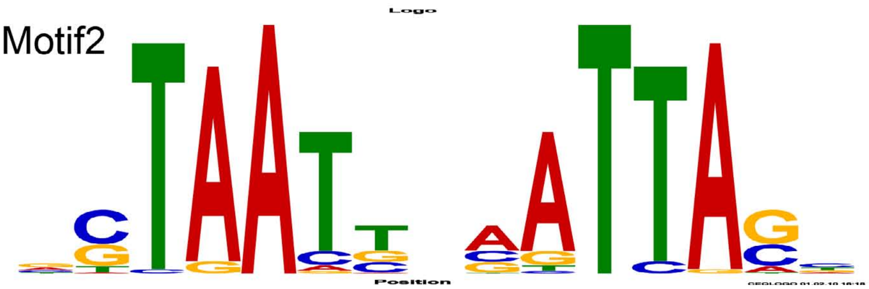

Motif2	(179 peaks)		
pvalue	1e-4	5e-4	1e-3
1000bp	4<br>3	13<br>17.57	27<br>37.06
500bp	2<br>0.98	3<br>6.49	9<br>14.55
200bp	0<br>0.32	1<br>1.72	2<br>4.57
100bp	0<br>0.11	0<br>0.91	0<br>2.1

Supplementary Figure S1: Predicted AT rich MIXL1 binding motifs are not enriched in the ChIP-seq Peaks. Motif1 and Motif2, previously characterized putative MIXL binding motifs [34–36], and randomized sequences of the same length were searched against the set of peaks (Supplementary Table 1) at various distances (1000bp, 500bp, 200bp, or 100bp as listed) from the peak apex, at either a *p*-value of $1e^{-4}$, $5e^{-4}$, or $1e^{-3}$. The first number in each cell denotes peaks that have the motif; the second number is the average of randomly shuffled sequences that have the random motif (the sequences were randomly shuffled 100 times to get the average). In all cases, the likelihood of finding the MIXL1 binding motif was not statistically significant or lower than the likelihood of identifying a random motif.

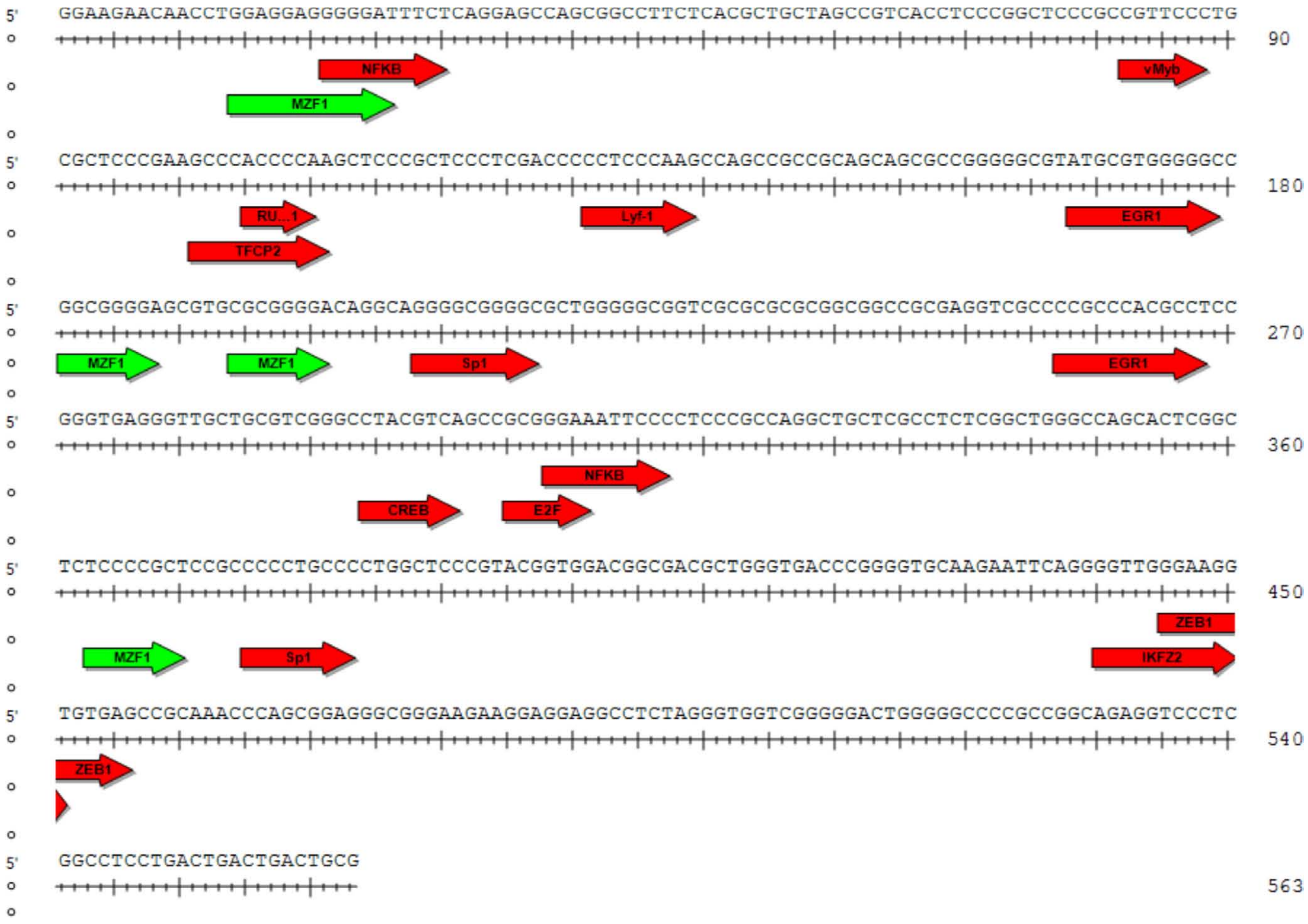

Supplementary Figure S2: Known Transcription Factor Binding Motifs in the *REL* Promoter. DNA sequence of the 550 bp *REL* promoter narrowed down by reporter assays is depicted. Transcription factor binding motifs by TFSearch are denoted. Of note, the most prevalent motif is the MZF1 zinc finger binding motif (denoted in green).

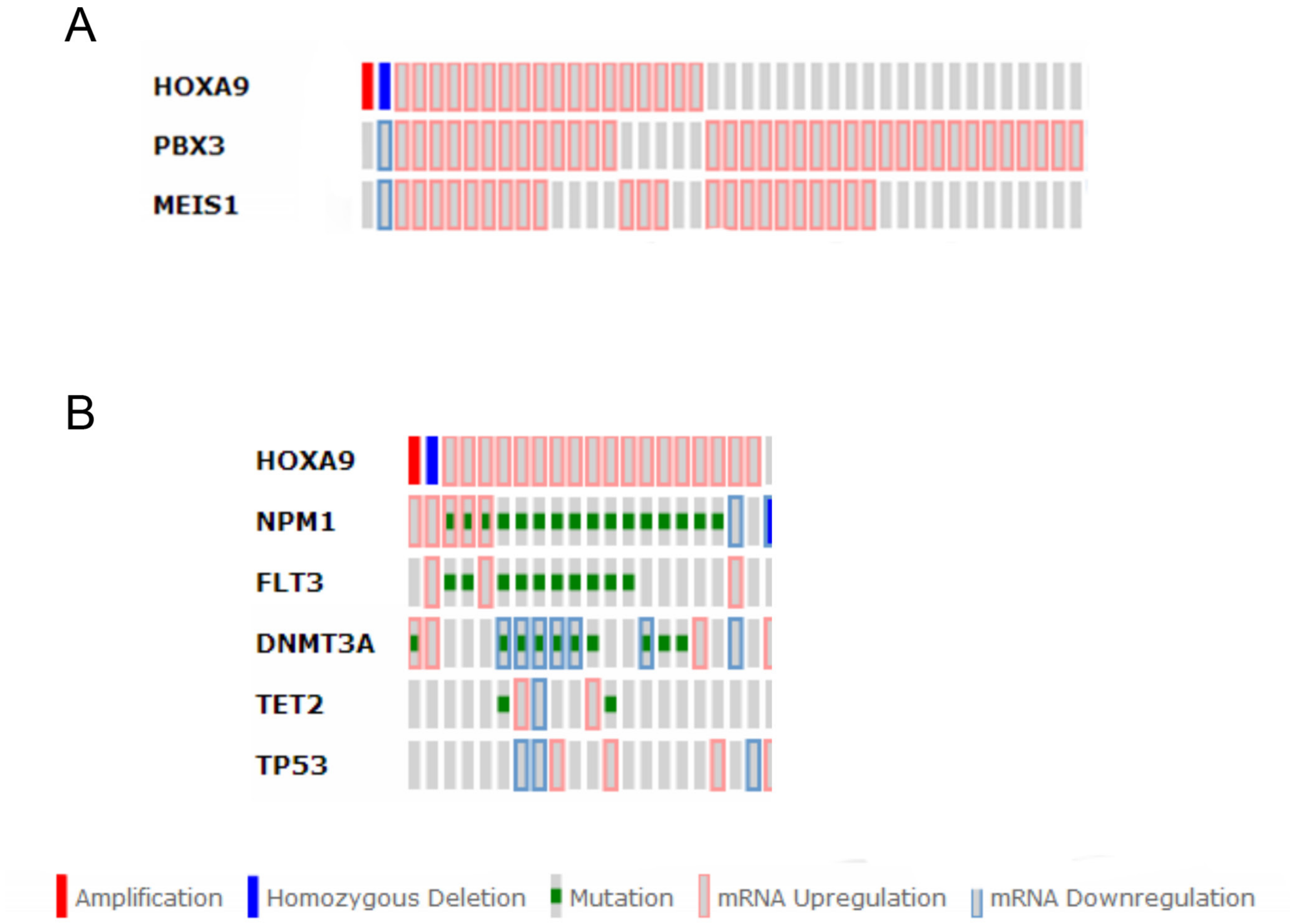

Supplementary Figure S3: *HOXA9* expression in the TCGA dataset is consistent with previous studies. Upper panel- Most *HOXA9* expressing patients have NPM1, FLT3 and DNMT3A mutations. Note the absence of *TP53* mutation in this subset. Lower panel- *HOXA9*, *PBX3* and *MEIS1* are frequently coexpressed.

CDX2

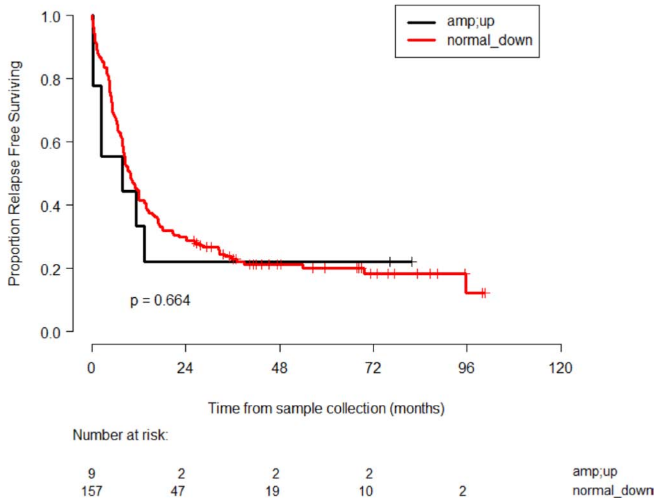

HLX

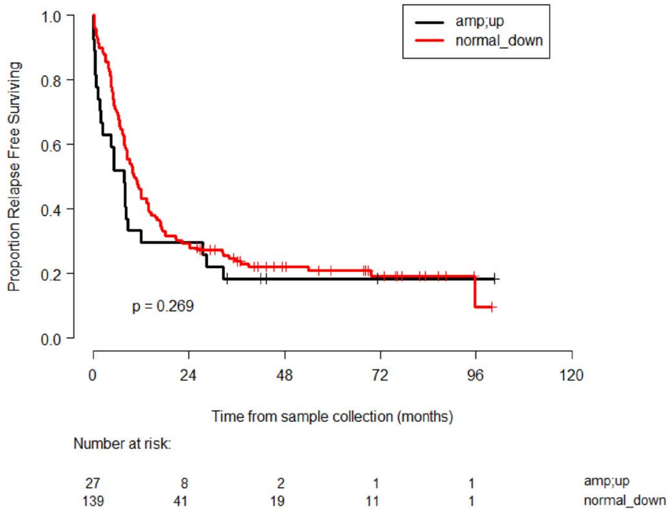

HOXA9

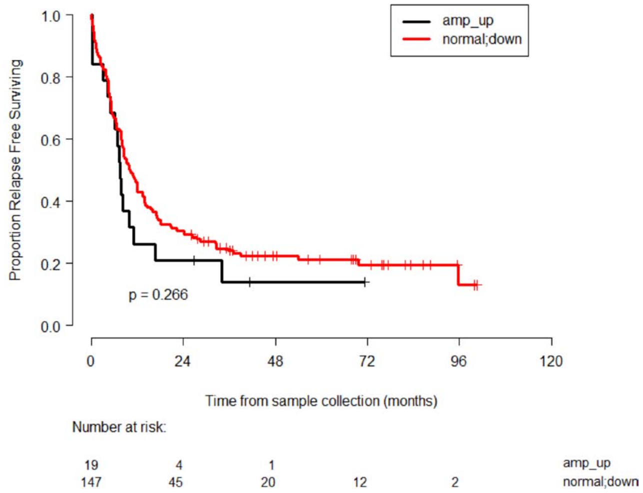

Supplementary Figure S4: (A-C) Relapse-free survival of *CDX2* (A), *HLX* (B) and *HOXA9* (C) expressing patients is not significantly different from the rest. Patients from the TCGA AML database were separated into two groups: >2 fold increase in expression or amplified *CDX2*, *HOXA9* and *HLX* and non-expressing or down regulated (*HLX*) cases. Each group was then compared based on Relapse-free survival data for using the Kaplan and Meier method.

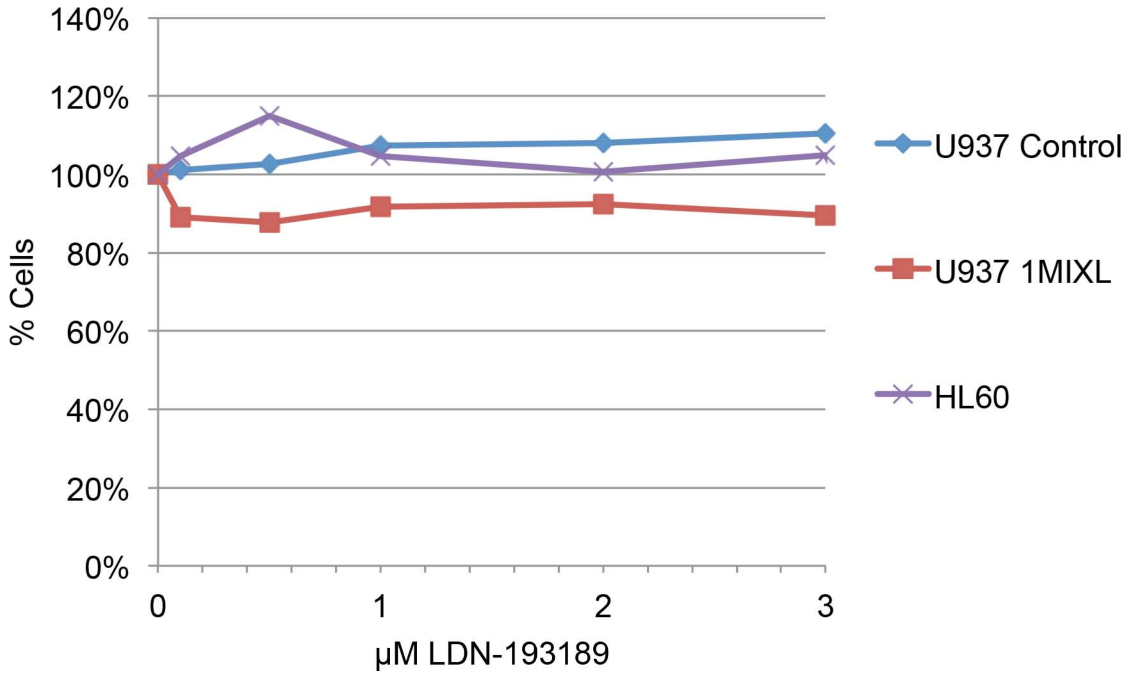

Supplementary Figure S5: U937 cells engineered to express *MIXL1* (1MIXL1) were not sensitive to LDN-193189. Each cell line was treated in triplicate with the indicated dosage of LDN-193189 with the drug or control medium replenished every 24 hours, for 4 days. Viability on day 4 was assayed by MTS assay. Absorbance was normalized to that of control samples treated with vehicle only.

Supplementary Table S2.

Gene Ontology Analysis: Gene IDs
**GO:0009987-Cellular Process**
NM_001037334, NM_138769, NM_016558, NM_020536, NM_001135672, NM_000363, NM_015263, NM_002908, NM_012388, NM_001001787, NM_014555, NM_017742, NM_012218, NM_024644, NM_002560, NM_019083, NM_031490, NM_020528, NM_025134, NR_027269, NM_004093, NM_024996, NM_017671, NM_004933, NM_021074, NM_194292, NM_007208, NM_003025, NM_001126050, NM_001039970, NM_016213, NM_025235, NM_001005741, NM_022466, NM_003375, NM_018127, NM_001042, NM_001033576, NM_152320, NM_022470, NM_005875, NM_003618, NM_006814, NM_001042681, NM_001184, NM_004804, NM_006590, NM_012335, NM_001609, NM_005804, NM_004960, NM_005801, NM_001039656, NM_004544, NM_017762, NM_001135175, NM_001039355, NM_006253, NM_005026, NM_001025778, NM_001102651, NM_001015053, NM_006086, NM_018981, NM_001128225, NM_001130141, NM_015508
**GO:0008135-Translation Factor activity, Nucleic Acid binding**
NM_019843, NM_024996, NM_005875, NM_005801, NM_015263
**GO:0043226-Organelle**
NM_019843, NM_138769, NM_016558, NM_020536, NM_001135672, NM_000363, NM_015263, NM_002908, NM_012388, NM_033542, NM_012218, NM_024644, NM_002560, NM_173852, NM_031490, NM_020528, NM_025134, NM_016070, NM_024996, NR_027128, NM_017671, NM_021074, NM_194292, NM_007208, NM_003025, NM_001126050, NM_016213, NM_025235, NM_001005741, NM_001136275, NM_022466, NM_003375, NM_018127, NM_001042, NM_152320, NM_022470, NM_001184, NM_001042681, NM_006590, NM_012335, NM_001609, NM_005804, NM_004960, NM_001039656, NM_004544, NM_001135175, NM_001039355, NM_006253, NM_001025778, NM_001102651, NM_001015053, NM_006086, NM_015602, NM_014329, NM_018981, NM_015508, NM_001130141, NM_022104
**GO:0044464-Cell Part**
NM_019843, NM_001037334, NM_138769, NM_152309, NM_016558, NM_020536, NM_001135672, NM_000363, NM_015263, NM_002908, NM_012388, NM_017849, NM_001001787, NM_001143839, NM_033542, NM_005898, NM_014555, NM_017742, NM_025058, NM_012218, NM_024644, NM_002560, NM_173852, NM_031490, NM_020528, NM_025134, NM_004093, NM_016070, NM_024996, NR_027128, NM_017671, NM_004933, NM_021074, NM_194292, NM_007208, NM_003025, NM_001126050, NM_001039970, NM_016213, NM_025235, NM_001005741, NM_001136275, NM_022466, NM_003375, NM_006133, NM_001011718, NM_018127, NM_001042, NM_152320, NM_022470, NM_001127395, NM_006814, NM_001042681, NM_001184, NM_006590, NM_001609, NM_012335, NM_005804, NM_005801, NM_004960, NM_001039656, NM_004544, NM_001135175, NM_012339, NM_001039355, NM_201544, NM_006253, NM_005026, NM_001025778, NM_001015053, NM_001102651, NM_006086, NM_015602, NM_014329, NM_018981, NM_001128225, NM_001130849, NM_001130141, NM_015508, NM_022104
**GO:0005623-Cell**
NM_138769, NM_016213, NM_025235, NM_022466, NM_003375, NM_001135672, NM_000363, NM_001042, NM_022470, NM_001184, NM_001042681, NM_033542, NM_001609, NM_012335, NM_012218, NM_004960, NM_024644, NM_004544, NM_002560, NM_173852, NM_001135175, NM_031490, NM_001039355, NM_025134, NM_001015053, NM_017671, NM_006086, NM_018981, NM_021074, NM_194292, NM_007208
**GO:0044422-Organelle Part**
NM_001039970, NM_001609, NM_012335, NM_025235, NM_005801, NM_003375, NM_024659, NM_173852, NM_015263, NM_031490, NM_012388, NM_016070, NM_017849, NM_001025778, NM_005875, NM_001015053, NM_006086, NM_014329, NM_018981, NM_007208, NM_005898

Supplementary Table S5.

	Peaks	Peaks with expression Change
Total	179	82
upstream	7	4
promoter	115	67
body	14	9
TSE	2	0
downstream	6	2
distant	35	0
